# Wright’s Principles of Vaccine Therapy*Read at annual meeting Seventh Councilor District Medical Society, Austin, Texas, December 19, 1907.

**Published:** 1908-01

**Authors:** H. N. Graves

**Affiliations:** Georgetown, Texas


### TEXAS MEDICAL JOURNAL.

Established July, 1885.

F. E. DANIEL, M. D., - - - - Editor, Publisher and Proprietor

PUBLISHED MONTHLY.—SUBSCRIPTION $1.00 A YEAR.

VOL. XXIII. AUSTIN, JANUARY, 1908. No. 7.

The publisher is not responsible for the views of contributors.

For the Texas Medical Journal.

### Wright's Principles of Vaccine Therapy.*

BY H. N. GRAVES, M. D., GEORGETOWN, TEXAS.

Wright's principles of vaccine therapy are founded on the knowledge of immunity and of the formation of immunizing substances in the body against the invasion of pathogenic or diseases producing bacteria.

Immunity is resistance to disease, and is classified as active and passive.

One is said to have active immunity to a certain infection when the cells of his own body have been stimulated to the production of the resisting or protective substances. Active immunity to some diseases, such as typhoid, scarlet fever, measles, etc., is obtained by recovery from the infection, or by the artificial stimulation of the body cells to produce the resisting or protective substances; for example, vaccination for smallpox.

Passive immunity may be obtained by injecting into the individual immunizing substances which have been derived from another animal; for example, diphtheria antitoxin from the body of a horse.

The process of immunization includes (a) leucocytes, which come into consideration in connection with the resistance to bacterial infection by virtue of the fact that they are capable of ingesting bacteria and of disintegrating these by intercellular digestion; (b) antibacterial elements of the blood fluid, these being

*Read at annual meeting Seventh Councilor District Medical Society, Austin, Texas, December 19, 1907.

"bacteriotropic" elements in the sense that they turn toward and enter into combination with the elements of the bacterial body.

The effect of the blood fluids on the bacterial body may kill the bacteria without dissolving them; they may be both killed and dissolved, or they may be so altered as to agglutinate in the presence of salt. Finally the bacteria may be so altered as to be readily ingested by the leucocytes.

The first two, viz., when the bacteria are killed with or without solution, are grouped as bactericidal and bacteriolytic effects respectively.

The substances in the blood which so alter bacteria as to render them readily ingested by the leucocytes are called opsonins by Wright. These opsonins are present in the circulating blood and tissue juices. Their identity is still under examination. They combine with bacteria and render them susceptible of being taken up and digested or destroyed by the leucocytes (phagocytes), hence the name opsonins from the Greek, opsono, "I convert into palatable pabulum."

Of the four varieties of the bacteriotropic substances the opsonins appear to be the most important.

The opsonins against any given infection may be increased by injecting subcutaneously small doses of the killed germs of the same species causing the infection.

The measure of the power of the blood-serum of an infected individual to prepare bacteria for ingestion by the leucocytes, compared with the serum from healthy individuals, is termed the "opsonic index." The opsonic index of healthy blood is taken as 1. For example, 100 leucocytes ingest 500 staphylococci treated with healthy serum. Using the serum of a person suffering with pustular acne, 100 leucocytes may take up only 400 staphylococci, the opsonic index of this person would be (500:400:1:0.8) 0.8.

For the determinations of the opsonic index, a measured quantity of a bacterial suspension is mixed thoroughly with equal amount of blood-serum (the opsonins being contained in the serum) and an emulsion of healthy leucocytes. This mixture is then incubated for several minutes to permit the opsonizing action of the serum on the bacteria and to allow the prepared organism to be ingested by the leucocytes. Slides are then made from the mixture and after staining, the number of bacteria in 100 leucocytes is counted.

Immediately after the injection of a bacterial vaccine, a period follows in which the opsonic index falls, due to a decrease of the opsonins in the serum. This is known as the "negative phase."

This is followed by a "positive phase," when there occurs an increase of opsonins in the serum. The opsonic index is above the normal and it is at this time that improvement is looked for in the symptom after the use of bacterial vaccine. It is possible to maintain this condition of high opsonic index by proper dosage of bacterial vaccines and careful spacing of intervals between doses.

It is therefore important to know when to administer bacterial vaccines for the purpose of maintaining a high opsonic index. It is manifest that the administration of the vaccine during the negative phase will still further lower the opsonic index, and that the injections, to produce therapeutic effects, must be administered during the "positive phase."

The existence of the "positive phase" may be ascertained either by taking the opsonic index or by noting the condition of the patient clinically. The negative phase is characterized by a "reaction" in which there is elevation of temperature, aggravation of the local symptoms, and a feeling of malaise. Not until the reaction is over is another injection to be given. The rule now being observed is to use doses sufficiently small not to produce a reaction, thus obviating the danger of giving doses during the "negative phase."

In regard to the site of injection, Wright says that every inoculation which is undertaken for therapeutic purposes deserves to be carefully considered in connection with the site of the focus of infection which is to be influenced. It will not suffice to inject the bacterial vaccine into any region of the subcutaneous tissue which may happen to present itself as convenient. The protective substances are elaborated in the tissue at the site of injection from which they find access to the blood channel. If the blood channel to which they find access does not lead to the focus of infection, the newly elaborated protective substances will not come into operation on that focus until they have been diluted by the whole volume of the blood. The inoculation, therefore, should be made "upstream" from the focus of infection, i. e., in some part of the lymph watershed which drains through the focus of infection. Unless the blood serum with its higher opsonic content is able to "get at" the local infection there can be no improvement although the opsonic index of the blood may be high.

When the bacteria from which the bacterial vaccine is prepared is obtained from infected individuals and intended to be injected into the individuals from whom the original cultures were obtained they are called "personal," "autogenous," or "identical" vaccines. When the vaccines are used in the treatment of similar infections occurring in other individuals they are called "stock" vaccines.

As the name vaccines more properly applied to Jenerian vaccination, the term "bacterins" has been suggested as more accurate descriptive names for bacterial vaccines. It has been found that "stock" vaccines or "stock" bacterins may be employed with equal benefit to autogenous vaccines in the treatment of certain infectious diseases, especially those due to staphylococci, gonococci, bacillus pyocyaneus, and bacillus tuberculosis.

Bacterial vaccines are contraindicated in acute diffused infections characterized by toxemia. The best results are obtained in subacute and chronic conditions. However, in some acute infections to be described later, bacterial vaccines have been very successfully employed.

The object in treating any infection with any bacterial vaccine is:

1. The production of a highly opsonized serum and lymph.
2. The bringing into contact the opsonized fluid with the infecting organism. For example, Wright and Douglas have demonstrated that the opsonic power of effusions is less than the circulating blood. Therefore, bringing into contact with bacteria and highly opsonized blood serum is the object of removing the exudate by tapping, purgation and other well known measures. This explains the beneficial results in the treatment of tubercular peritonitis by laparotomy and the removal of pleuritic exudates.

In abscess there exists another factor, viz., a ferment which not only destroys the leucocytes but also destroys the opsonins of the surrounding blood. The object in the opsonic treatment of an old suppurating abscess is to destroy bacteria by providing a highly opsonized blood serum, and at the same time to safeguard the tissues and the leucocytes from the digestive power of the ferment by replacing the pus by highly opsonized lymph from the circulating blood. This is accomplished by draining off the pus in a suitable manner, and applying substances which attract the flow of the lymph and at the same time reduce the coagulability of the blood and thus maintaining the supply of opsonized blood.

The conditions obtaining in sinuses are alike those which prevail in an abscess which is discharging without emptying itself; there is a pus-fluid possessing low opsonic power, which exerts a digestive effect on the tissues, and makes itself manifest by the sodden, unhealthy appearance of the surrounding skin. The obstacles to the inflow of lymph in a choked sinus are the density of the granulation tissue, and the lining membrane of fibrin. For the dislodgment of bacteria from the walls of such a dry sinus something more is needed than a mere increase of the opsonic power of the blood and circulating lymph.

As above stated, a deficient flow of lymph and the formation of a lining of fibrin of its walls are favorable to the survival of microbes in the sinus. These conditions may be combated by introducing into every dry sinus a solution of 0.5 per cent of citrate of soda and 10 per cent sugar (or in lieu thereof a solution of 0.5 per cent citrate of soda and 5 per cent sodium chloride), supplementing this treatment when necessary by Klapp's system of dry cupping.

The citrate of soda prevents coagulation and scabbing by decalcifying the lymph, and the sugar or salt, acting by osmosis causes fluids to transude from the blood vessels. Under the influence of these applications the clear lymph begins to well out from the previously choked sinuses and the local conditions rapidly improve.

Autoinoculations follow all active and passive movements which affect a focus of infection and all vascular changes which activate the lymph stream in such a focus.

In discussing the comparative merits of treatment by induced autoinoculation of bacterial vaccines, Wright calls attention to the fact that we have in spontaneous autoinoculation, in artificially induced inoculation, and in the inoculation of bacterial vaccines, the three great agencies by which immunizing responses are evoked in the organism. He states with confidence that the immunizing effects which are achieved by autoinoculation are far more expensive in the respect that the patient obtains a much smaller yield of protective substances, for an equivalent of intoxication; and he calls attention to the fact that there is no risk of disseminating living bacteria in the organism if bacterial vaccines are employed instead of autoinoculation because they are sterilized. He then goes on to point out in detail the disadvantages of autoinoculation as compared with the advantages to be obtained from bacterial vaccines.

Wright says that the question as to whether vaccine therapy can or can not be employed in septicemic diseases is a question which ought to be approached without bias and one which can be decided only by experiment. In his opinion bacteriotropic substances are manufactured locally by the tissues, and, obviously, the conditions for successful immunization will be less favorable when the vaccination elements are thrown into the circulation of the blood than when they are inoculated directly into the tissues. For, in the latter cases, they will come into application on the tissues in a concentrated form, which will not be the case if these substances are diluted by the whole volume of blood. It is, therefore, rational to assume that, from the point of view of his immunization, such a patient might derive advantage from the inoculation of bacterial vaccines.

Wright summarizes briefly his experience of the practical results of vaccine therapy, commencing with the type of bacterial infection which presents the simplest therapeutic problems.

First. *Penetration of Single Species of Micro-organisms in the Body.*—Typical examples are (1) when tubercle bacilli have effected a lodgment in lymphatic glands, (2) when staphylococci have penetrated into the subcutaneous tissue, causing as yet only suppurative (furuncular) as distinguished from necrotic (carbuncular) changes.

In Wright's experience, all but unifomly successful results have been achieved by vaccine therapy, in furunculosis within a few days, in tuberculous infections of the lymphatic gland, between five weeks and eighteen months—on the average of about six months. The same observation applies to tubercular infections of the testicle and to simple tubercular infections of the kidney and urinary passages, also (reservedly on account of the author's restricted experience) to early cases of tubercle of the lung.

Second. *Ulcerative Type of Infection.*—This type is met in connection with the breaking down of nodules in the deeper tissues, or with the penetration of superficial infections into the deeper tissues. Except when secondary infections have supervened this type is as tractable to vaccine therapy as the one just mentioned. If anything, an open ulcer uncomplicated by secondary infection is more tractable than a localized infection in the deeper tissues. An ulcer which starts from the surface ultimately penetrates to the lymph-bearing strata, and, when it has done so, the lymph wells up through the floor of the ulcer and becomes affected with the micro-organism present.

Third. *Infections of the Skin.*—In their relations to bacterial therapy affections of the skin may be divided in two classes: Dry, scaly, non-vascular, and the vascular. The former is extremely intractable and the latter very tractable, according to the author's opinion founded on his own experience. A typical example of the former is furnished by the superficial scaly form of lupus (so-called lupus psoriasis); of the latter pustular acne and pustular staphylococcal sycosis are examples.

Fourth. *Infections of the Mucous Membrane and of the Glands and Ducts Connected Therewith.*—In the author's experience such infections are readily influenced by vaccine therapy. He had obtained evidence or had experience of successful results in many different infections of the mucous lining of the ear, antrum, nose, nasal sinuses, dental alveoli, and salivary glands, also bacillus coli infection of the intestinal mucous membrane and gall bladder, and in many different infections of the uterus, urinary bladder, and urethra. In case of bladder infections there is generally a bacteriuria. The extinction of the infection of the mucous membrane is not necessarily always followed by the cessation of the bacteriuria, although in five of the author's cases this was the result.

Fifth. *Infections of Sinuses.*—Successful results were obtained by the author when the inoculations from bacterial vaccines were combined with the course of treatment referred to above under the head removal of obstacles to the free-streaming of lymph to a focus of infection.

Sixth. *Mixed Infections.*—In the author's opinion the suggestion that mixed infection must necessarily be considered in every case of phthisis, lupus, tubercular caries, tubercular cystitis, and tubercular ulceration, will not be acceptable to many clinicians, however, whether acceptable or unacceptable, there is no escape from the fact that practically every case of suppurating lupus is complicated by staphylococcus infection. And what is true of lupus is also true of every tubercular affection to which microbes can find access.

Having called attention to the magnitude and far-reaching nature of the tissue involved in the treatment of mixed infections, Wright considered the question of the results achieved by vaccine therapy. These he divided as follows:

First. *Case in Which Vaccine Therapy is Directed to the Destruction of Only One of the Infecting Microbes.*—In regard to cases in which vaccine therapy is directed to the destruction of only one of the infecting microbes, the employment of vaccine therapy directed to the destruction of a single species of microbes it usually leaves the other species quite unaffected. In exceptional cases, the extinction of one of the microbes under the influence of the corresponding vaccine has indirectly led to the extinction of the other. Sometimes—and this applied in particular to surface infections of mucous membranes or ulcers—vaccine therapy directed to the destruction of one infecting microbes conduces to the multiplication of the competing microbes.

Second. *Case in Which Vaccine Therapy is Directed to the Destruction of all Infecting Microbes.*—Successful results have been obtained, notably in lupus, cystitis, and endometritis, when in cases of mixed infection measures were taken to immunize the patient against each of the different infections. The patient does not find the task of responding to a series of different vaccines, administered in appropriate and properly interspaced doses, more difficulty than the task of responding to one variety of vaccine only.

Third. *Generalized Infections.*—In association with his fellow workers, Wright had, up to the date of his lecture, treated by vaccine therapy some half dozen cases of Malta fever and an equal number of cases of streptococcal septicemia. The Malta fever cases were favorably influenced. In the streptococcal septicemia cases a complete cure was achieved in a case of malignant endocarditis.

